# PLCA8 suppresses breast cancer apoptosis by activating the PI3k/AKT/NF‐κB pathway

**DOI:** 10.1111/jcmm.14578

**Published:** 2019-08-26

**Authors:** Misha Mao, Yongxia Chen, Yunlu Jia, Jingjing Yang, Qun Wei, Zhaoqing Li, Lini Chen, Cong Chen, Linbo Wang

**Affiliations:** ^1^ Department of Surgical Oncology, Sir Run Run Shaw Hospital Zhejiang University Hangzhou China

**Keywords:** breast cancer, PLAC8, tumour growth

## Abstract

The cysteine‐rich lysosomal protein placenta‐specific 8 (PLAC8), also called onzin, has been shown to be involved in many types of cancers, and its role is highly dependent on cellular and physiological contexts. However, the precise function of PLAC8 in breast cancer (BC) progression remains unclear. In this study, we investigated both the clinical significance and biological functions of PLAC8 in BC progression. First, high PLAC8 expression was observed in primary BC tissues compared with adjacent normal tissues through immunohistochemistry analysis. The results of in vitro and in vivo assays further confirmed that PLAC8 overexpression promotes cell proliferation and suppress BC cell apoptosis, whereas PLAC8 silencing has the opposite effect. In addition, the forced expression of PLAC8 greatly induces cell migration, partially by affecting the EMT‐related genes, including down‐regulating E‐cadherin expression and facilitating vimentin expression. Further mechanistic analysis confirmed that PLAC8 contributes to cell proliferation and suppresses cell apoptosis in BC by activating the PI3K/AKT/NF‐κB pathway. The results of our study provide new insights into an oncogenic role of PLAC8 and reveal a novel PLAC8/ PI3K/AKT/NF‐κB pathway as a potential therapeutic target for BC.

## 1 INTRODUCTION

Breast cancer (BC) is the most common malignant tumour and the second leading cause of cancer‐related death in women.^1^, ^2^, ^3^ Thus, further investigation into the underlying mechanisms of BC progression is urgently needed.

Placenta‐specific 8 (PLAC8, onzin) was originally identified as being highly expressed in the mouse placenta.^4^ Subsequent studies have shown that PLAC8 is involved in regulating metabolism and immunity and participates in cancer pathophysiological processes, such as cell proliferation, differentiation, apoptosis and autophagy.^5^, ^6^, ^7^, ^8^, ^9^, ^10^ The results of several studies have revealed that PLAC8 affects cell growth by modulating the phosphatidylinositol 3‐kinases (PI3K)/protein kinase B(AKT) signalling pathway in hepatocellular carcinoma. Furthermore, in nasopharyngeal carcinoma, PLAC8 contributes to radioresistance by inhibiting the PI3K/AKT/GSK3β pathway, as knockout of PLAC8 sensitizes nasopharyngeal carcinoma cells to radiation by activating the PI3K/AKT/GSK3β pathway. These intriguing findings elucidated pivotal oncogenic or tumour suppressor roles for PLAC8 in cancer progression. However, the potential mechanisms and functional role of PLAC8 in BC pathogenesis remain unknown. Apoptosis is considered to be the major mechanism of programmed cell death, and the maintenance of normal physiology and cellular homeostasis occurs through different mechanisms.^11^, ^12^, ^13^ Apoptosis regulates diverse cellular processes, including cell proliferation, the cell cycle and survival,^14^, ^15^ and the results of previous studies have suggested that the inhibition of PI3K/AKT signalling induces apoptosis.^16^, ^17^

In this study, we aimed to determine the expression profile and elucidate the pathological functions of PLAC8 during BC progression, and our results revealed a relationship between endogenous PLAC8 expression and PI3K/AKT pathway activity. PLAC8 knockdown in Bcap‐37 cells induced apoptosis (ie cell growth inhibition), and improved cell growth in T47d cells was observed as a result of PLAC8 overexpression. Herein, we demonstrate that PLAC8 acts as a significant factor in BC progression by altering the activity of the PI3k/AKT/NF‐κB pathway.

## 2 MATERIALS AND METHODS

### 2.1 Tissue specimens

Fifty‐five paired BC and adjacent tissues samples were obtained from the Sir Run Run Shaw Hospital (affiliated with Zhejiang University) that had been histopathologically and clinically diagnosed from 2012 to 2018. The tumour grade and metastasis were measured according to the American Joint Committee on Cancer, 8th Edition. All tissues were frozen and stored in liquid nitrogen until analysed. All patients gave informed consent to use excess pathological specimens for research purposes. The study was approved by the Ethics Committee of the Sir Run Run Shaw Hospital, which is affiliated with Zhejiang University.

### 2.2 Cell lines and culture

Human BC cell lines (HCC1937, T47D, MCF‐7, Hs‐578T, SK‐BR‐3, BT549, BCAP‐37 and MDA‐MB‐231) were purchased from the American Type Culture Collection (ATCC) and stored in liquid nitrogen. The human BC cell lines (HCC1937, T47D, BT549 and BCAP‐37) were maintained in RPMI 1640 Medium supplemented with 10% foetal bovine serum and 5% glutamine. MCF‐7 and Hs‐578T cells were maintained in Dulbecco's modified Eagle's medium supplemented with 10% foetal bovine serum and 0.01 mg/ml bovine insulin. SK‐BR‐3 cells were maintained in McCoy's 5A Medium supplemented with 10% foetal bovine serum. MDA‐MB‐231 cells were maintained in Leibovitz's L‐15 Medium supplemented with 10% foetal bovine serum. All cell lines were cultured at 37°C, and with the exception of MDA‐MB‐231, they were grown in a humidified atmosphere containing 5% CO_2_. LY294002 was purchased from Selleck (S1105, Texas, USA).

### 2.3 Transfection

Short interfering RNAs targeting PLAC8 (Si‐PLAC8) and a scrambled control siRNA were designed and purchased from RiboBio (Guangzhou, China). The Si‐PLAC8 sequences were CCTTGGGTGTCAAGTAFCA (Si‐PLAC8#1) and GGAACAAGCGTCGCAATGA (Si‐PLAC8#2). Lentiviral PLAC8‐overexpressing and negative control vectors were gifts from Dr Yongxia Chen. Cells were transfected with siRNAs or plasmids in serum‐free medium using Lipofectamine 3000 (Invitrogen, USA) according to the manufacturer's instructions.

### 2.4 Western blot analysis

Cells were lysed with RIPA lysis buffer supplemented with 1× PMSF. Whole‐cell lysates were separated by SDS‐PAGE (Bio‐Rad, Berkeley, CA, USA) and transferred to PVDF membranes (Millipore, Billerica, MA, USA). Subsequently, the blots were blocked with 5% non‐fat milk for 1 hour. The primary antibodies (1:1000 dilution) used in this study were as follows: PLAC8 (Cell Signaling Technology, Boston, Massachusetts, USA #13885), PI3K P85 (Cell Signaling, #13666), pPI3K (Tyr485/Tyr199) (Cell Signaling, #4228), AKT (Cell Signaling, #4691), pAKT‐Ser473 (Cell Signaling, #4060), pAKT‐Thr308 (Cell Signaling, #13038), NF‐κB p65 (Cell Signaling, #8242), pNF‐κB (Cell Signaling, #3033), mTOR (Cell Signaling, #2983), pGSK‐3b (Cell Signaling, #5558), PARP (Cell Signaling, #9542), caspase‐3 (Cell Signaling, #9662), cleaved caspase‐3 (Cell Signaling, #9664), caspase‐8 (Cell Signaling, #9746), caspase‐9 (Cell Signaling, #9502), Bcl‐2 (Cell Signaling, #15071), E‐cadherin (Cell Signaling, #3195) and vimentin (Cell Signaling, #5741). GAPDH (1:1000) antibodies were purchased from Santa Cruz Biotechnology (L0910, CA, USA). The blots were probed with primary antibody at 4°C overnight and then incubated with the secondary antibody (Abcam, Cambridge, MA, USA) for 1 hour at room temperature. Goat anti‐mouse IgG H&L (ab6708) and goat anti‐rabbit IgG H&L (ab6721) were purchased from Abcam. Reactive bands were visualized with ECL Plus reagents using a LAS‐4000 mini instrument. The total protein band intensities were normalized to the loading control (GAPDH) and qualified using ImageJ.

### 2.5 Immunohistochemical staining

Slices of paraffin‐embedded tissues were deparaffinized and rehydrated in xylene and graded alcohol solutions and then blocked with 3% H_2_O_2_ for 5 minutes and 3% bovine serum albumin (Roche, Hong Kong, China) for 15 minutes. The slices were subsequently stained with PLAC8 (1:200), Ki‐67 (1:500) (100130‐MM22, Sino Biological, Beijing, China) and cleaved caspase‐3 (1:1000) antibodies for 1 hour at 37°C. The tissue slices were subsequently washed with PBS three times for 3 minutes each and then stained with the secondary antibody from the GT Vision III Immunohistochemical Assay kit (GK500710, Gene Tech, Shanghai, China) according to the manufacturer's instructions. The results of the staining assay were evaluated and scored independently by two pathologists who were unaware of the clinical outcome. The staining for PLAC8 was graded with four scores: strong, +3; moderate, +2; weak, +1; and absent, 0. Specimens with scores of +3 or +2 were defined as having high expression, and those with scores of +1 or 0 were defined as having low expression. All images were captured using a fluorescence microscope (Olympus BX‐51, Japan).

### 2.6 Immunofluorescence staining

Cells were briefly seeded onto glass coverslips in 24‐well plates until reaching 50%‐60% confluence. Cells were washed 3 times and blocked in PBST (PBS containing 0.1% Tween) supplemented with 2.5% bovine serum albumin (Sigma‐Aldrich, St Louis, USA) at room temperature for 30 minutes. Subsequently, the cells were washed 3 times and then incubated with the PLAC8 antibody (1:500) at 37°C for 1 hour, followed incubation with an Alexa 488‐conjugated (green) goat anti‐rabbit antibody (1:1000) (70‐GAR4881; Multisciences, Hangzhou, China) to detect the target protein. DAPI was used to visualize the nuclei of the cells, and images were acquired using a Nikon laser scanning confocal microscope (Nikon Instruments Inc, Melville, NY, USA).

### 2.7 Cell proliferation assay

Cells (0.7 × 10^4^) were seeded into a 96‐well culture plate for 12, 24 or 72 hours. Cell viability was evaluated using an MTT assay (CellTiter 961 AQueous One Solution Cell Proliferation Assay, Promega). The absorbance was measured at 490 nm using a BioTek ELx800 absorbance microplate reader.

### 2.8 Wound‐healing assay

Cells (5 × 10^5^) were seeded into 6‐well plates and incubated until reaching 80%‐90% confluence. Scratch wounds were made using a pipette tip 48 hours after transfection. Subsequently, the cells were washed with PBS 3 times to remove cell debris and then were incubated in complete medium. The scratch was recorded under a phase‐contrast microscope at the time of wound generation (0 hour) and at 24 hours. The gap widths were measured using ImageJ.

### 2.9 Transwell migration and invasion assays

For the Transwell (Corning Costar, Cambridge, MA, USA) migration assays, cells were collected in medium without serum. Cell invasion was measured using Transwell chambers coated with Matrigel (Corning Costar, Cambridge, MA, USA). Cells (5 × 10^4^) transfected with an siRNA or plasmid in 100 µL of medium without serum were transferred into the upper chamber of the Transwell, and 600 μL of medium containing 10% FBS was added to the lower chamber. After 24 hours of incubation, cells on the upper surface of the membrane were carefully removed using a cotton swab. The membrane was then fixed with 4% paraformaldehyde and stained with 0.5% crystal violet solution for 15 minutes, after which images were captured under a microscope (Zeiss, Primovert).

### 2.10 Apoptosis and cell cycle flow cytometry

Cells were seeded into 6‐well plates and then collected after 48 hours of transfection with an siRNA or plasmid. The number of apoptotic cells was determined using a BD Annexin V‐FITC/PI Assay kit according to the manufacturer's instructions. For cell cycle analysis, cells were harvested and washed 3 times, and the cell cycle distribution of cells was determined using a Cycle Staining kit (CCS012, Multisciences, Hangzhou, China) according to the manufacturer's instructions. Cell apoptosis and cell cycle analyses were performed by flow cytometry (Accuri model C6).

### 2.11 TUNEL apoptosis assay

TUNEL apoptosis assays were performed with a KeyGEN Biotech kit (KGA7052, Jiangsu, China) according to the manufacturer's protocol. The cells were seeded into 6‐well plates and cultured for 24 hours. Following transfection for 48 hours, the cells were collected, and the cells or frozen slides of nude mice were blocked with 3% H_2_O_2_ in methanol. A sufficient volume of proteinase K was added to completely cover the cells or tissue, and the samples were incubated for 30 minutes in a humidified chamber at room temperature. After washing the slides three times with PBS for 5 minutes, 50 μL of TdT reaction buffer was added to each slide, and the slides were incubated for 1 hour at room temperature in the dark. The cells or slides were subsequently washed with PBS three times for 5 minutes each, after which 50μl of streptavidin‐FITC reaction buffer was added to each slide. The cells or slides were then incubated for 30 minutes at room temperature in the dark, and then, images were acquired using a Nikon laser scanning confocal microscope (Nikon Instruments Inc, Melville, NY, USA).

### 2.12 RNA isolation and quantitative real‐time PCR

Total RNA was extracted from cells and tissues using TRIzol reagent (Invitrogen, Carlsbad, CA) according to the manufacturer's instructions. Total cDNA was synthesized using a HiFiScript cDNA Synthesis kit (CW2596M, CWBIO, Jiangsu, China). Quantitative real‐time PCR was performed with ChamQ SYBR qPCR Master Mix (Q411‐02, Vazyme, Nanjing, China) in an ABI 7300 instrument (Applied Biosystems Inc, USA). The conditions for reactions were as follows: 95°C for 30 seconds; 40 cycles of 95°C for 10 seconds; and 60°C for 30 seconds, followed by incubations at 95°C for 15 seconds, 60°C for 1 minutes, 95°C for 15 seconds and 60°C for 15 seconds. The expression of mRNA was assessed by evaluating the threshold cycle (CT) values, with GAPDH mRNA used as an endogenous control. Relative expression was calculated using the relative quantification equation (RQ) = 2‐ΔΔCt. BLAST searches with the primers and melting curve analyses were performed to ensure the specificity of amplification. Experiments were performed 3 times independently, and gene expression was normalized to that of GAPDH. The following primers were used: GAPDH forward, 5’‐TGACTTCAACAGCGACACCCA‐3’; GAPDH reverse, 5’‐CACCCTGTTGCTGTAGCCAAA‐3’; PLAC8 forward, 5’‐GGAACAAGCGTCGCAATGAG‐3’; and PLAC8 reverse 5’‐AAAGTACGCATGGCTCTCCTT‐3’.

### 2.13 Tumour xenografts in nude mice

Twelve BALB/c nude mice (aged 4‐6 weeks, from Shanghai Laboratory Animal Center, Shanghai, China) were housed in a specific pathogen‐free environment. According to the expression of the target genes, nude mice were randomly divided into two groups: Control (with empty vector) or PLAC8 (overexpressing PLAC8) (n = 6). T47D cells (2 × 10^6^) transfected with a PLAC8‐overexpressing lentivirus or empty vector in 100μl of PBS with 100μl of growth factor‐reduced basement membrane matrix (Corning Costar, Cambridge, MA, USA) were injected into the right subaxillary region of each mouse. The tumour size was measured using a slide calliper twice per week, and the tumour volume was calculated using the following formula: (A × B^2^)/2 (A, the length of the tumour; B, the width of the tumour). Eighteen days after injection, the mice were killed, and the subcutaneous growth of each tumour was examined. The wet tumour weight was calculated as the mean weight ± standard deviation (SD) for each group. This study was approved by the Ethics Committee for Animal Studies of Zhejiang University (Hangzhou, China).

### 2.14 Statistical analysis

Chi‐square tests were used to evaluate PLAC8 expression and the clinicopathological features of BC. Comparisons between multiple groups were performed with multiple comparisons by one‐way ANOVA. Student's *t* test was used for pairwise comparisons. All data were obtained from at least three independent experiments. The values are presented as the means ± SD *, *P* < 0.05; **, *P* < 0.01; NS, not significant. All analyses were performed with GraphPad Prism 7.0 (San Diego, CA, USA).

## 3 RESULTS

### 3.1 PLAC8 expression is frequently up‐regulated in breast cancer

**Figure 1:**
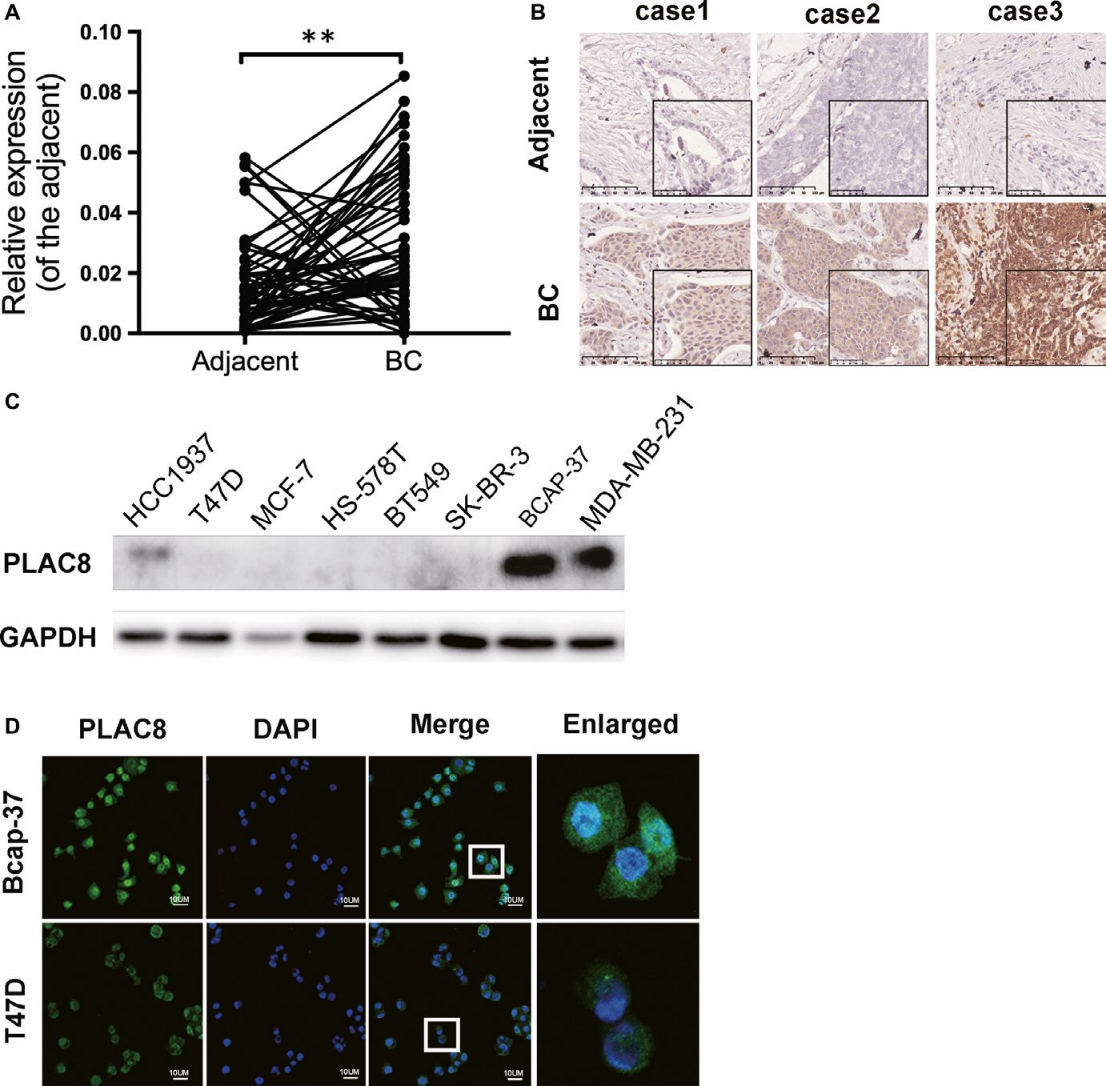
PLAC8 is relatively up‐regulated in breast cancer. A, The mRNA expression of PLAC8 is increased in breast cancer tissues compared with adjacent tissues, as determined by qPCR. The mRNA expression was normalized to adjacent tissues. The error bars correspond to the means ± SD. (B) IHC staining showed the expression of PLAC8 in breast cancer and adjacent tissues of patients. 20× and 40×; the scale bar represents 100 and 50 μm, respectively. C, The PLAC8 protein expression levels of breast cancer cell lines (HCC1937, T47D, MCF‐7, Hs‐578T, SK‐BR‐3, BT549, BCAP‐37 and MDA‐MB‐231). D, The subcellular localization of PLAC8 in BCAP‐37 and T47D cells was detected by immunofluorescence analysis. An enlarged view of the boxed area from each group confirms the presence of PLAC8‐positive cells. The scale bar represents 10 μm. The values are presented as the means ± SD ^**^ *P* < 0.01

To date, the results of several studies have strongly suggested that PLAC8 regulates cell division, differentiation and apoptosis.^9^, ^18^, ^19^ To determine the biological function of PLAC8 in BC, we first evaluated the level of PLAC8 mRNA in 55 samples of BC and their corresponding adjacent tissues. The level of PLAC8 mRNA was increased in BC tumour tissues (Figure 1A). Furthermore, we analysed the IHC staining results for the samples and showed that PLAC8 was up‐regulated in the cancer tissues compared with the adjacent tissues (Figure 1B). In addition, PLAC8 was observed to be closely correlated with tumour size and TNM stage (Table 1, Table S1). These findings suggest that PLAC8 may contribute to BC progression. In addition, we assessed the expression of PLAC8 protein in various BC cell lines and observed that PLAC8 expression was relatively higher in Bcap‐37 and MDA‐MB‐231 cells than in the other cells assayed (Figure 1C). Unsurprisingly, the expression of PLAC8 was dramatically different in various BC cell lines. We further confirmed the subcellular location of PLAC8 using immunofluorescence staining with a PLAC8‐specific antibody, the results of which showed that PLAC8 was primarily labelled in the cytoplasm (Figure 1D). Taken together, these data strongly indicate that PLAC8 is associated with BC progression.

**Table 1:** The relationship between PLAC8 expression and clinicopathological characteristics in breast cancer

Clinicopathological characteristics	PLAC8 expression(n = 55)		*P*‐value
	Low	High	
Age(years)			
⩽50	16	17	0.565
>50	11	11	
Tumour size(cm)			
⩽2	15	8	0.043*
>2	12	20	
Lymph node metastasis			
No	17	11	0.068
Yes	10	17	
TNM stage			
I	12	4	0.005**
II	14	15	
III	1	9	

* *P* < 0.05

** *P* < 0.01

### 3.2 Overexpression of PLAC8 enhances cell viability and migration in breast cancer

**Figure 2:**
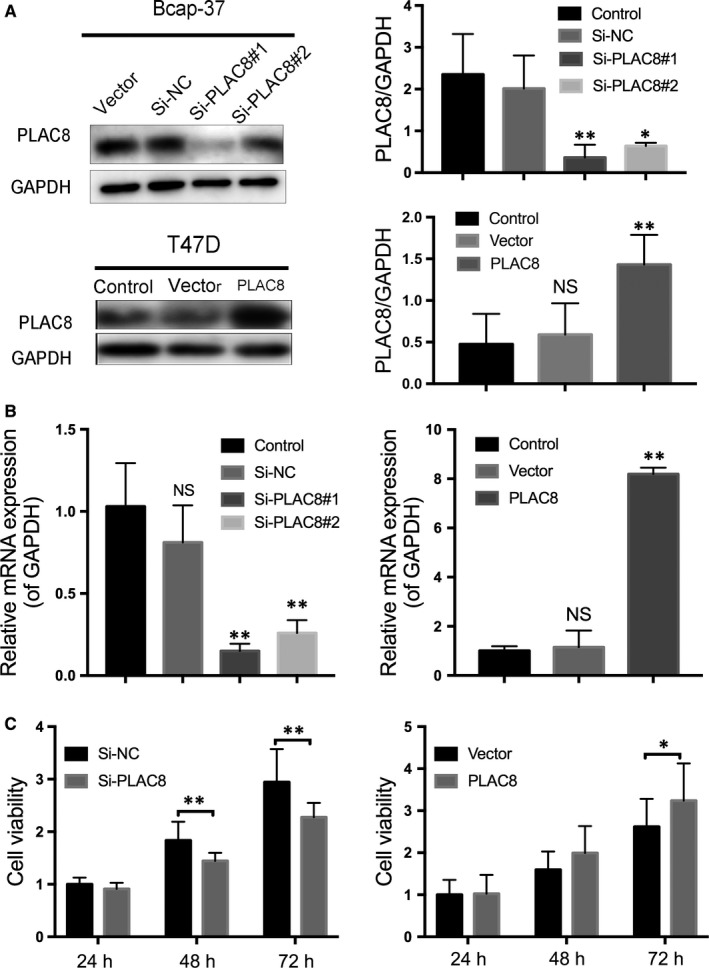
PLAC8 promotes cell proliferation *in vitro*. A and B, Bcap‐37 and T47D cells were transfected with PLAC8 siRNA and/or the PLAC8 overexpression plasmid, and the interference effect of siRNA or plasmid was determined by Western blotting (upper) 72 h after transfection and qRT‐PCR (bottom) 48 h after transfection. The expression was quantified and normalized to GAPDH. The error bars correspond to the means ± SD. (C) Cell proliferation assays were performed to determine the cell viability for Bcap‐37 and T47D cells transfected with PLAC8 siRNA or plasmid. The error bars correspond to the means ± SD. The values are presented as the means ± SD ^*^ *P* < 0.05; ^**^ *P* < 0.01; NS, not significant

**Figure 3:**
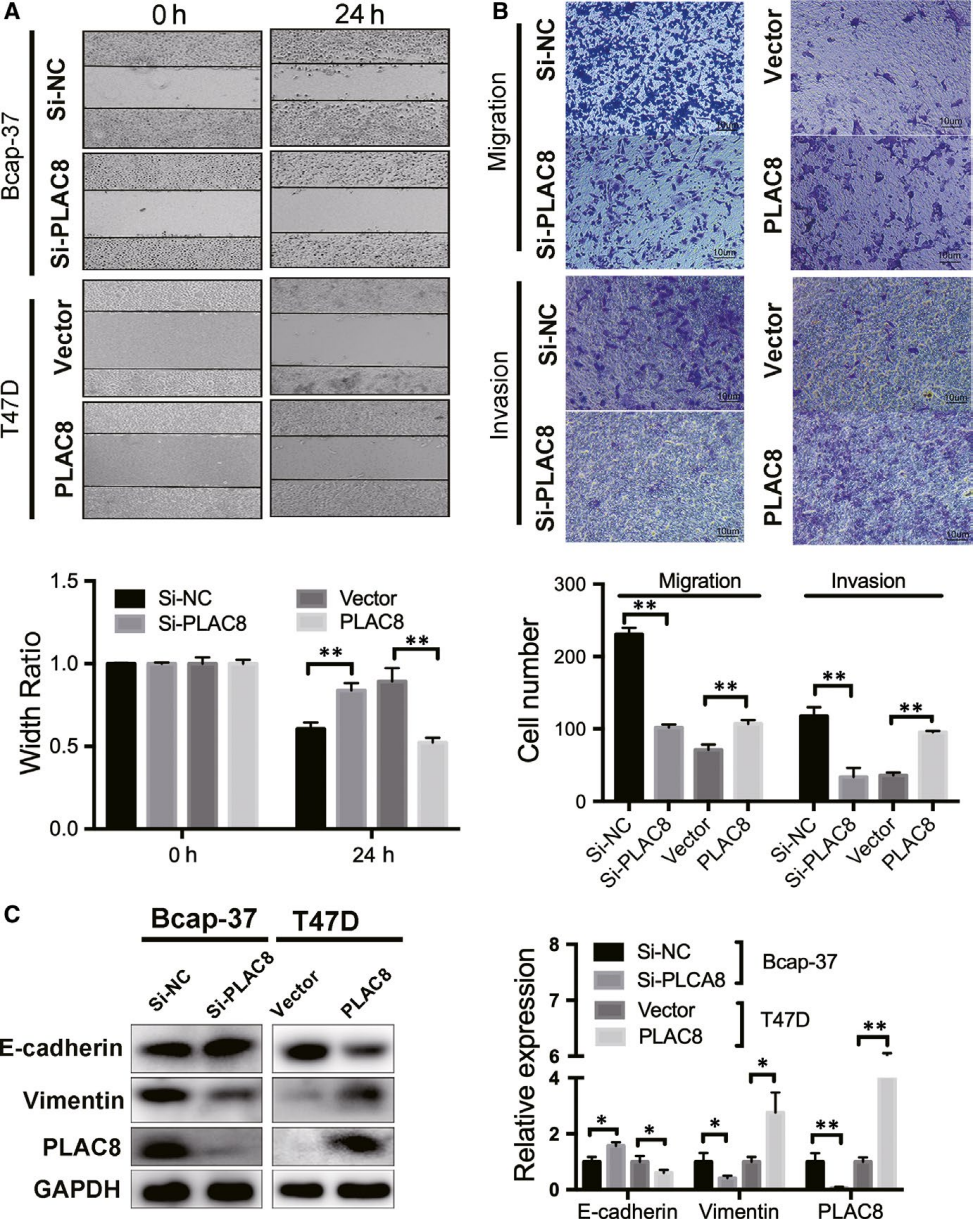
PLAC8 knockdown significantly inhibits cell migration and invasion and suppresses the EMT phenotype. A, Wound‐healing assays were conducted in Bcap‐37 or T47D cells transfected with PLAC8 siRNA or the PLAC8 overexpression plasmid. Migration distance was measured at 0 and 24 h after cells were scratched, Bcap‐37 Si‐NC or T47D vector cells were used as controls. The error bars correspond to the means ± SD. B, Representative images show that cell migration (upper) and invasion (bottom) increased in the PLAC8‐overexpressing cells. The error bars correspond to the means ± SD. C, Western blot analysis of the relative expression of EMT‐related markers in Bcap‐37 or T47D cells 72 h after transfection. The expression was quantified and normalized to GAPDH. The error bars correspond to the means ± SD. The values are presented as the means ± SD ^*^ *P* < 0.05; ^**^ *P* < 0.01; NS, not significant

Because PLAC8 expression was relatively higher in Bcap‐37 cells, and T47D cells exhibited a loss of PLAC8 expression, we chose Bcap‐37 and T47D cells for subsequent experiments. Western blot and qRT‐PCR analyses were performed to confirm the altered PLAC8 in Bcap‐37 cells transfected with PLAC8 siRNA (Si‐PLAC8#1 and Si‐PLAC8#2) or non‐targeting control (Si‐NC) and T47D cells transfected with the PLAC8 or vector plasmids (Figure 2A,B). As shown in Figure 2A,2, we used Si‐PLAC8#1 as Si‐PLAC8 in our latter experiments. The results of cell proliferation assays revealed that PLAC8 silencing inhibited Bcap‐37 cell growth. In contrast, PLAC8 overexpression enhanced cell viability in T47D cells (Figure 2C). The results of wound‐healing and Transwell assays showed that PLAC8 silencing significantly reduced the migration and invasion abilities of BC cells (Figure 3A,B). To determine whether PLAC8 affected cell migration and invasion via the epithelial‐mesenchymal transition (EMT) pathway, we analysed the expression of EMT‐related markers by Western blotting and observed an increase in vimentin expression in cells with up‐regulated PLAC8 expression, whereas E‐cadherin expression was decreased by PLAC8 overexpression (Figure 3C). Thus, PLAC8 was demonstrated to promote cell proliferation as well as the migratory and invasive ability of BC cells.

### 3.3 PLAC8 silencing induces caspase 3/9 activation, Bcl‐2 up‐regulation and apoptosis of breast cancer cells

**Figure 4:**
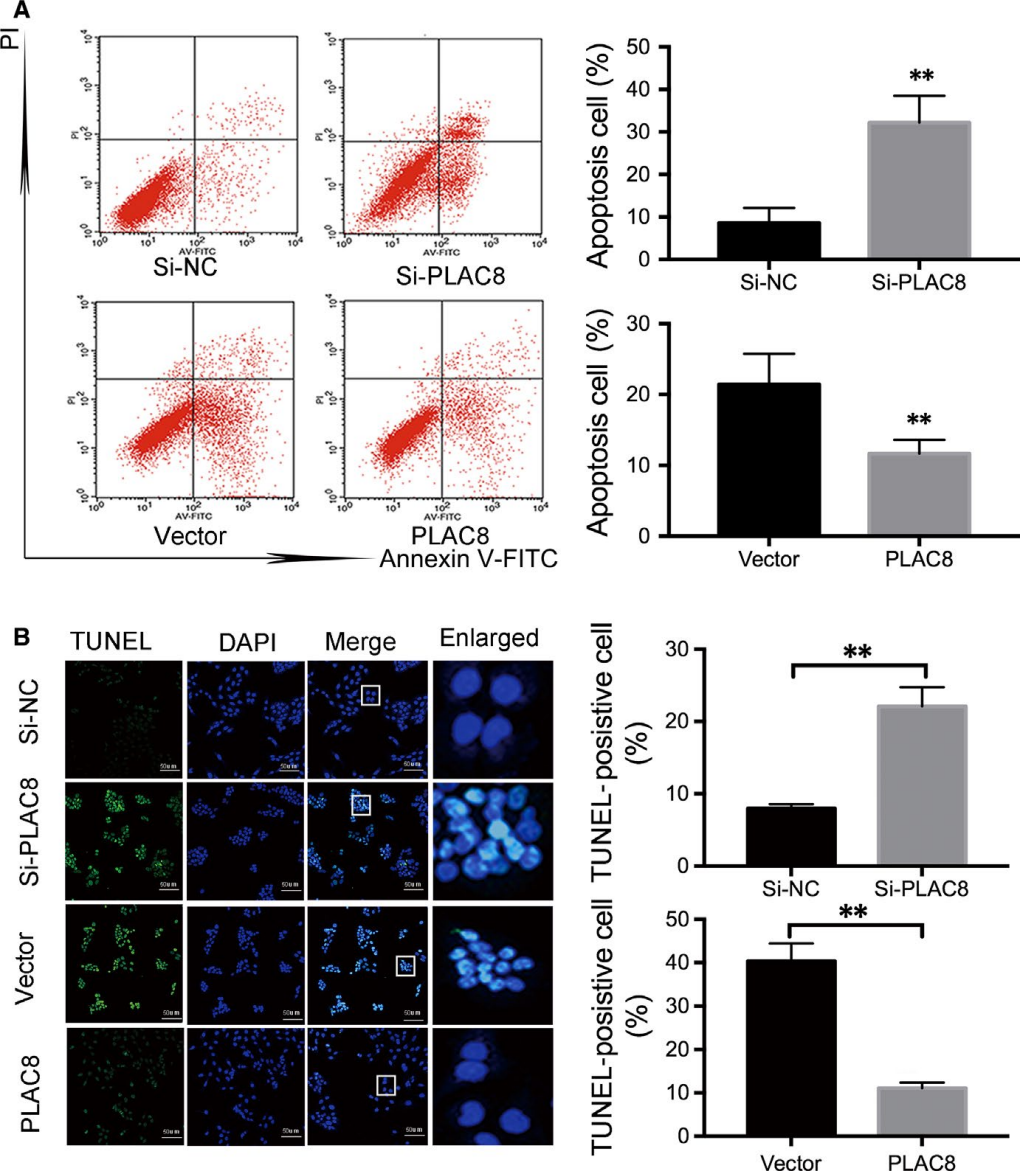
PLAC8 inhibits apoptosis both in vivo and vitro. A and B, Bcap‐37 and T47D cells were stained with Annexin V/PI or Tunnel solution, and the percentage of apoptotic cells was determined using a flow cytometer 48 h after transfection. An enlarged view of the boxed area from each group confirms the presence of TUNEL‐positive cells. The values are presented as the means ± SD ^**^ *P* < 0.01; NS, not significant

**Figure 5:**
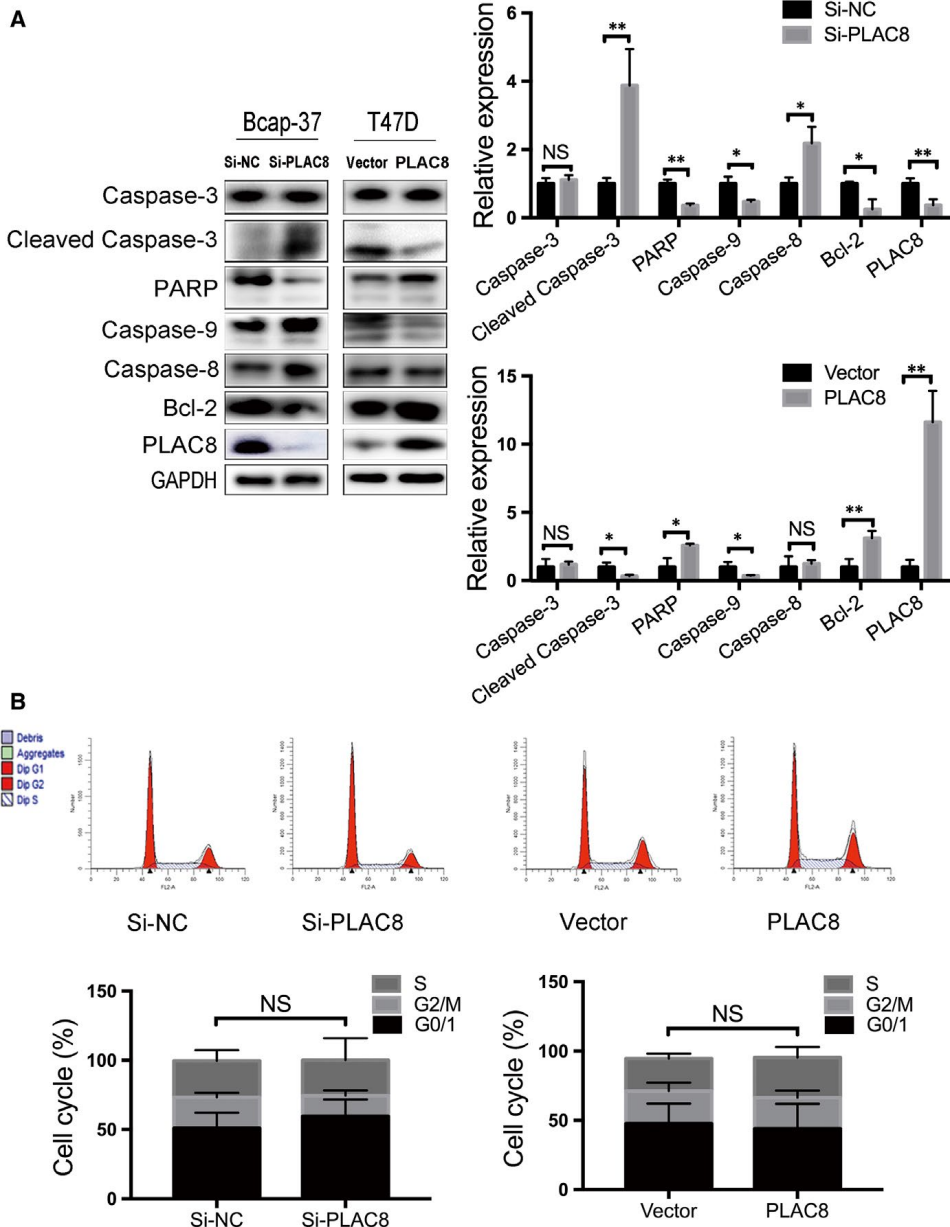
PLAC8 inhibits cell proliferation by influencing apoptosis in vivo. A, Western blot analysis of the relative expression of apoptosis‐related markers 72 h after transfection. The expression was quantified and normalized to GAPDH. The error bars correspond to the means ± SD. B, Analysis of the cell cycle in Bcap‐37 or T47D cells 48 h after PLAC8 siRNA or plasmid transfection, determined using a flow cytometer. The error bars correspond to the means ± SD. The values are presented as the means ± SD ^*^ *P* < 0.05; ^**^ *P* < 0.01; NS, not significant

**Figure 6:**
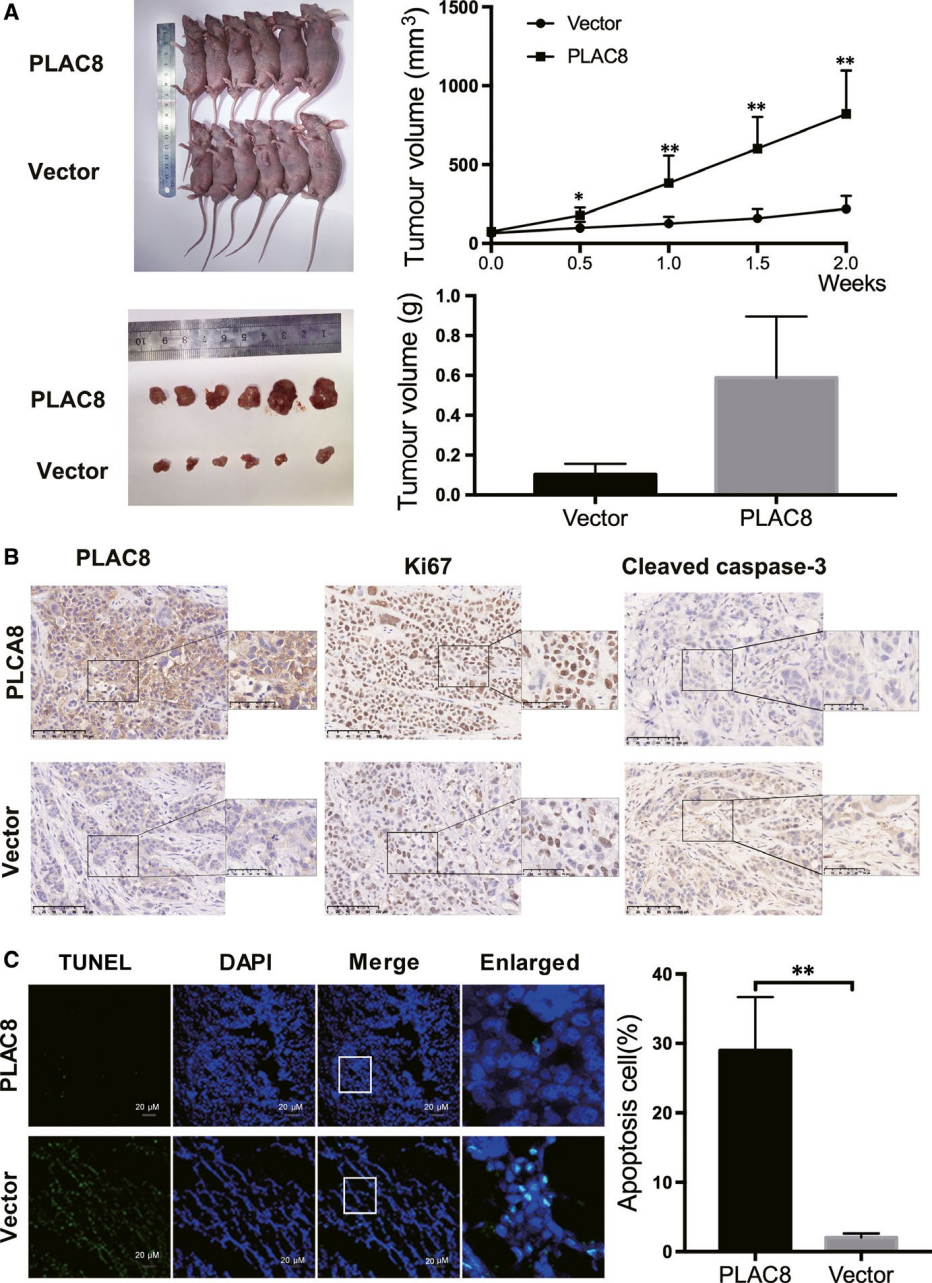
PLAC8 enhances tumour growth in vitro and in vivo by decreasing apoptosis. A, Tumour volume (measured twice a week) and tumour weight (day 18) were compared between the PLAC8‐overexpressing and vector groups, which showed tumour volumes and weights that were comparable to those observed in the vector group. The tumour volume was calculated using the following equation: V = (width^2^ × length)/2. The error bars correspond to the means ± SD. B, IHC staining showed the expression of PLAC8, Ki67 and cleaved caspase‐3. 20× and 40×; the scale bar represents 100 and 50 μm, respectively. C, Assessment of apoptosis by TUNEL assays of tumours from mice in the PLAC8 overexpression and vector groups on day 18. The scale bar represents 20 μm. An enlarged view of the boxed area from each group confirms the presence of TUNEL‐positive cells. The values are presented as the means ± SD ^*^ *P* < 0.05; ^**^ *P* < 0.01; NS, not significant

Recent studies have shown that the aberrant regulation of apoptosis results in uncontrolled cell proliferation.^20^, ^21^, ^22^ As PLAC8 overexpression facilitates BC cell proliferation, we assessed whether increased or decreased PLAC8 expression contributes to cell apoptosis to influence BC progression. The results revealed that PLAC8‐transfected T47D cells were inhibited for apoptosis, whereas PLAC8 knockdown significantly induced apoptosis in Bcap‐37 cells (Figure 4A,B). Furthermore, we determined the expression of apoptosis‐related markers by Western blotting (Figure 5A). Anti‐apoptotic (Bcl‐2) and pro‐apoptotic (cleaved caspase‐3) proteins play crucial roles in controlling cells apoptosis.^23^, ^24^, ^25^, ^26^ Significant increases in cleaved caspase‐3 and decreased Bcl‐2 expression were observed in Bcap‐37 cells transfected with PLAC8 siRNA, whereas T47D cells overexpressing PLAC8 exhibited the opposite effect. Furthermore, the cell cycle analysis results indicated that more Bcap‐37 cells transfected with si‐PLAC8 were in the G0/1 phase than that observed for the control cells (Figure 5B), and these results were consistent with those observed in T47D cells. However, increased or depleted expression of PLAC8 did not significantly influence cell cycle progression. These data suggest that PLAC8 expression is positively correlated with cell growth and is negatively correlated with the apoptosis of BC cells. In addition, we observed that tumours in nude mice from the PLAC8‐overexpressing groups grew significantly faster and larger than those from the control groups at 18 days post‐implantation (Figure 6A). The IHC staining results for Ki67, PLAC8 and cleaved caspase‐3 as well as the TUNEL assay results showed increased tumour proliferation and significantly decreased apoptosis in the PLAC8‐overexpressing groups (Figure 6B,C). Taken together, these results suggest that PLAC8 can regulate BC proliferation by inhibiting cell apoptosis in vitro and in vivo.

### 3.4 PLAC8 suppresses cell apoptosis via the PI3k/AKT/NF‐κB pathway

**Figure 7:**
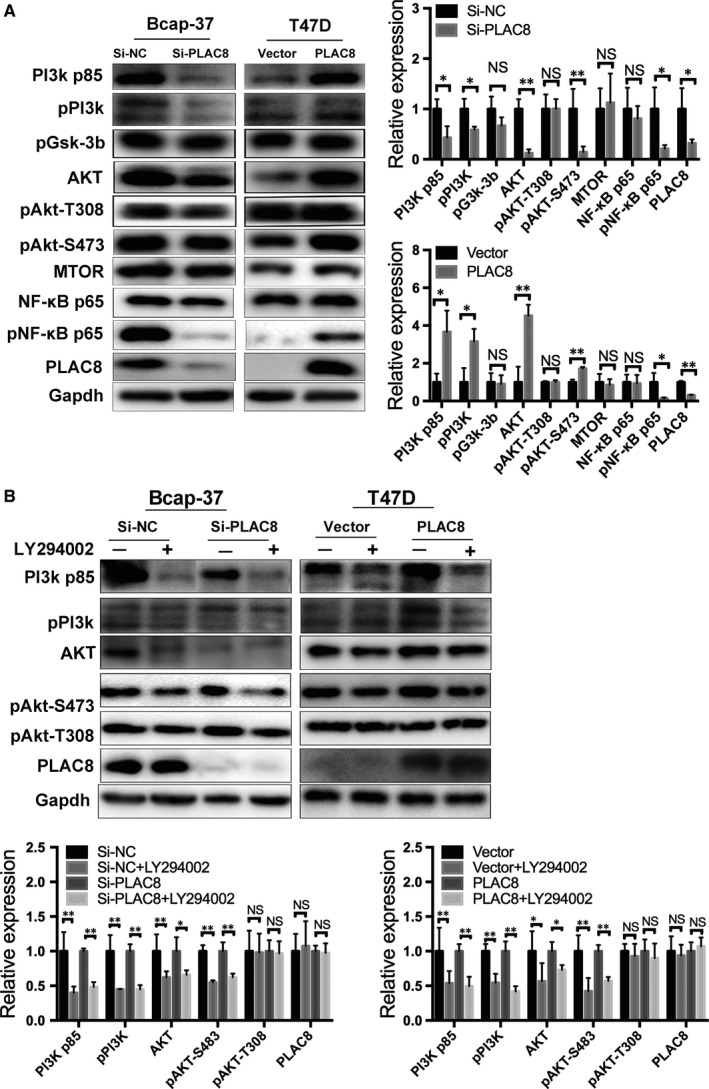
PLAC8 knockdown induces apoptosis and affects cell proliferation by inhibiting the PI3K/AKT/NF‐κB pathway. A, Western blot analysis of Bcap‐37 or T47D cells 72 h after PLAC8 siRNA or plasmid transfection. Scramble RNA or vector plasmid was used as negative controls. The levels of PI3K/AKT/NF‐κB pathway‐related markers were measured. Protein expression was quantified and normalized to GAPDH. The error bars correspond to the means ± SD. (B) Cells were transfected for 48 h and subsequently cultured with or without LY294002 (10 μm) for 24 h. Western blot analysis showing changes in the levels of PI3K pathway‐related markers. Protein expression was quantified and normalized to GAPDH. The error bars correspond to the means ± SD. The values are presented as the means ± SD ^*^ *P* < 0.05; ^**^ *P* < 0.01; NS, not significant

We speculated that PLAC8 regulates BC cell apoptosis in vitro and in vivo by regulating the expression of different target genes. To further elucidate the mechanisms by which PLAC8 inhibits BC cell apoptosis, we examined the putative upstream regulators and downstream targets of PLAC8. The PI3K/AKT pathway not only plays a central role in cell cycle distribution, survival and drug sensitivity but also is associated with cell growth and apoptosis.^17^, ^27^, ^28^, ^29^ Because these properties make the PI3K/AKT pathway a major candidate for investigating cell proliferation, we aimed to examine the potential relationship between PLAC8 and the PI3K/AKT pathway in BC. To determine whether PLAC8 regulates apoptosis via this pathway in BC cells, the levels of PI3K/AKT pathway‐associated molecules and their phosphorylated forms were examined. The results of Western blot analyses showed that the levels of PI3K p85, pPI3K, AKT, pAKT‐s473 and pNF‐κB p65 were significantly higher in T47D cells overexpressing PLAC8 (Figure 7A). In addition, changes in the expression of PI3K p85, pPI3K, AKT and pAKT‐s473 in response to PLAC8 significantly were attenuated by LY294002, a strong inhibitor of phosphoinositide 3‐kinases (PI3Ks) (Figure 7B). Taken together, these results indicate that PLAC8 regulates the apoptosis of BC cells through the PI3K/AKT/NF‐κB pathway.

## 4 DISCUSSION

The results of our study provide direct evidence for the pro‐tumorigenic role of PLAC8 in BC. BC tissues exhibited high PLAC8 expression compared with that observed in normal tissues. In addition, the overexpression of PLAC8 enhanced tumour growth in vitro and in vivo, as demonstrated by the observed increase in the invasive and migration capacities and decreased apoptosis induction in tumour cells, whereas PLAC8 silencing significantly inhibited cell growth. Further integrated analyses provided insights into the underlying oncogenic role of PLAC8 in BC, as PLAC8 modulation of the PI3k/AKT/NF‐κB signalling pathway was shown to be necessary to induce cell apoptosis. Collectively, the tumour‐promoting role of PLAC8 through its regulation of the PI3k/AKT/NF‐κB signalling pathway has not been previously reported.

Breast cancer is the most common cancer among women. To improve the rate of diagnosis in the early stages of BC and develop effective therapies, it is necessary to review all possible resources related to BC processes. PLAC8 is a 115‐amino acid, cysteine‐rich protein,^30^ and accumulating evidence suggests that PLAC8 has vital roles in many important cellular processes and in the progression of several types of cancer. Interestingly, PLAC8 exerts different biological effects depending on the cellular context.^7^, ^31^ As a regulatory protein, PLAC8 binds to the promoter and induces the transcription of C/EBPb, which is involved in brown fat differentiation, thermoregulation and the control of body weight.^5^ The overexpression of PLAC8 has been shown to up‐regulate the activation of Rac1 and Cdc42, resulting in human interstitial extravillous trophoblast cell invasion and migration.^10^ Furthermore, PLAC8 was shown to be associated with transcriptional and/or post‐translational modification of the central cell cycle regulators CDKN1A, retinoblastoma protein and cyclin D1 (CCND1), promoting pancreatic cancer cell growth.^9^ However, in hepatocellular carcinoma, PLAC8 is a tumour suppressor that is regulated by miR‐185‐5p, which suppresses cell proliferation.^32^ The precise role and underlying mechanism of action of PLAC8 in various cancers, especially BC, remain unclear, and our results further our understanding of the cellular functions of this protein in BC.

In this study, we observed that the inhibition of PLAC8 expression suppressed the tumorigenic and metastatic capacity of BC by attenuating the PI3k/AKT/NF‐κB axis. First, we analysed several clinicopathological factors, including BC subtypes, age, tumour size, lymphatic metastasis and tumour grade, which are related to the risk of BC tumour progression, in 55 BC tissue samples. PLAC8 was observed to be up‐regulated in cancer tissues compared with its expression in adjacent tissues. Patients with high levels of PLAC8 in BC tissues commonly show larger tumour sizes and higher TNM stages. To further confirm this transformation, we performed cell proliferation, migration and invasion assays as well as apoptosis analyses and xenograft tumour growth studies. Our in vitro and in vivo observations were similar to those that have been made in patients. These results strongly suggest that PLAC8 has a potential role in BC tumorigenesis. The PI3K/AKT pathway is one of the most frequently activated downstream signal transduction pathways in human cancer. Thus, PI3K/Akt pathway‐related transduction, including its mechanisms of activation, signal‐transducing molecules and effects on gene expression, contributes to tumorigenesis. Abnormal activation of the PI3k/AKT pathway is observed in numerous solid tumours, including BC,^17^, ^33^, ^34^, ^35^ and this pathway controls a series of cellular processes, including cell proliferation, cell cycle, apoptosis, autophagy and cell migration. For example, miR‐613 acts as an upstream regulator that disturbs the interaction between YAP and WBP2, influencing the activity of the EGFR/PI3K pathway in triple‐negative BC cells.^34^ Similarly, cPLA2α has been shown to activate PI3k/Akt signalling to mediate the TGF‐β‐induced EMT in BC cells.^36^ PI3K has been linked to an extraordinarily diverse group of cellular functions via the activation of Akt. In addition, AKT phosphorylates a host of cellular proteins, including GSK3β, mTOR and NF‐κB, to facilitate cell survival and apoptosis.^37^, ^38^, ^39^ To confirm whether PLAC8 regulates BC proliferation through the PI3K pathway, we analysed the expression of PI3k/AKT/NF‐κB pathway markers, the results of which were consistent with the observed changes in PLAC8 expression. We have been suggested that there may be a significant relationship between PLAC8 and the PI3k/AKT pathway with respect to BC progression. Our results demonstrated that inhibition of the PI3k/AKT pathway is negatively correlated with the altered expression of PLAC8, as the repression of PLAC8 inhibited the PI3k/AKT/NF‐κB axis. Thus, the present study emphasizes the association between PLAC8 and the PI3K‐mediated pathway cascade in BC progression and highlights its possible usefulness for therapeutic intervention.

In summary, in this study, we showed that PLAC8 is an oncogene that can actively promote cell viability and proliferation in vitro and in vivo by acting as an upstream regulator that activates the PI3K/Akt/NF‐κB pathway in BC. Taken together, these results reveal a novel role for PLAC8 in tumorigenesis and provide novel insights into the molecular pathogenesis of BC.

## CONFLICTS OF INTEREST

The authors have no conflicts of interest to declare.

## AUTHOR CONTRIBUTIONS

Linbo Wang and Misha Mao designed the study; Misha Mao, Yongxia Chen and Yunlu Jia performed the experiments; Misha Mao, Qun Wei and Jingjing Yang analysed and interpreted the data; Misha Mao, Yongxia Chen and Yunlu Jia wrote the manuscript. All authors read and approved the final manuscript.
