# Supplementary material for: PLCA8 suppresses breast cancer apoptosis by activating the PI3k/AKT/NF‐κB pathway
Source: J Cell Mol Med. 2019 Aug 26;23(10):6930–41. doi: 10.1111/jcmm.14578 (PMC6787500; doi:10.1111/jcmm.14578)
Supplement: Supplementary file 1 [file JCMM-23-6930-s001.docx]

Table S1

Supplementary information of 55 patients in Table 1

| Case | Age | TNM | Metastasis |
| --- | --- | --- | --- |
| 1 | 36 | T_1_N_2_M_0_ | No |
| 2 | 47 | T_2_N_2_M_0_ | No |
| 3 | 41 | T_2_N_1_M_0_ | No |
| 4 | 42 | T_2_N_1_M_0_ | No |
| 5 | 46 | T_2_N_0_M_0_ | No |
| 6 | 70 | T_2_N_0_M_0_ | No |
| 7 | 71 | T_2_N_0_M_0_ | No |
| 8 | 55 | T_1_N_0_M_0_ | No |
| 9 | 48 | T_1_N_0_M_0_ | No |
| 10 | 48 | T_1_N_1_M_0_ | No |
| 11 | 45 | T_2_N_0_M_0_ | No |
| 12 | 52 | T_1_N_0_M_0_ | No |
| 13 | 50 | T_1_N_0_M_0_ | No |
| 15 | 60 | T_2_N_1_M_0_ | No |
| 15 | 48 | T_1_N_0_M_0_ | No |
| 16 | 44 | T_2_N_1_M_0_ | No |
| 17 | 55 | T_2_N_1_M_0_ | No |
| 18 | 62 | T_2_N_0_M_0_ | No |
| 19 | 40 | T_3_N_0_M_0_ | No |
| 20 | 42 | T_2_N_0_M_0_ | No |
| 21 | 65 | T_1_N_1_M_0_ | No |
| 22 | 47 | T_1_N_0_M_0_ | No |
| 23 | 64 | T_1_N_1_M_0_ | No |
| 24 | 63 | T_1_N_1_M_0_ | No |
| 25 | 48 | T_1_N_0_M_0_ | No |
| 26 | 75 | T_1_N_0_M_0_ | No |
| 27 | 45 | T_2_N_2_M_0_ | No |
| 28 | 45 | T_2_N_1_M_0_ | No |
| 29 | 41 | T_3_N_2_M_0_ | No |
| 30 | 50 | T_2_N_1_M_0_ | No |
| 31 | 64 | T_2_N_2_M_0_ | No |
| 32 | 59 | T_2_N_1_M_0_ | No |
| 33 | 48 | T_1_N_0_M_0_ | No |
| 34 | 43 | T_1_N_0_M_0_ | No |
| 35 | 67 | T_1_N_0_M_0_ | No |
| 36 | 46 | T_2_N_1_M_0_ | No |
| 37 | 59 | T_2_N_1_M_0_ | No |
| 38 | 44 | T_2_N_2_M_0_ | No |
| 39 | 77 | T_1_N_0_M_0_ | No |
| 40 | 49 | T_1_N_0_M_0_ | No |
| 41 | 73 | T_3_N_1_M_0_ | No |
| 42 | 58 | T_1_N_1_M_0_ | No |
| 43 | 55 | T_3_N_0_M_0_ | No |
| 44 | 52 | T_2_N_0_M_0_ | No |
| 45 | 45 | T_3_N_1_M_0_ | No |
| 46 | 41 | T_2_N_1_M_0_ | No |
| 47 | 47 | T_2_N_2_M_0_ | No |
| 48 | 42 | T_1_N_0_M_0_ | No |
| 49 | 43 | T_1_N_0_M_0_ | No |
| 50 | 46 | T_2_N_0_M_0_ | No |
| 51 | 50 | T_2_N_1_M_0_ | No |
| 52 | 47 | T_1_N_0_M_0_ | No |
| 53 | 52 | T_2_N_2_M_0_ | No |
| 54 | 42 | T_1_N_0_M_0_ | No |
| 55 | 58 | T_2_N_0_M_0_ | No |
